# Plasmid Genomic Dynamics and One Health: Drivers of Antibiotic Resistance and Pathogenicity

**DOI:** 10.3390/pathogens14101054

**Published:** 2025-10-18

**Authors:** Célia P. F. Domingues, João S. Rebelo, Francisco Dionisio, Teresa Nogueira

**Affiliations:** 1 cE3c—Centre for Ecology, Evolution and Environmental Changes & Change, Global Change and Sustainability Institute, Faculdade de Ciências, Universidade de Lisboa, 1749-016 Lisboa, Portugalfadionisio@fc.ul.pt (F.D.); teresa.nogueira@iniav.pt (T.N.); 2 INIAV—National Institute for Agrarian and Veterinary Research, 2780-157 Oeiras, Portugal

**Keywords:** plasmids, one health, genomics, horizontal gene transfer, plasmid classification, antibiotic resistance genes, virulence genes, multidrug resistance, bacterial evolution

## Abstract

Seen through a One Health perspective, plasmids act as global links, connecting human, animal, and environmental microbiomes while broadening the ecological scope of resistance and virulence. By combining knowledge about plasmid classification, mobility, resistance, virulence, and data sources, this review emphasizes their key role as drivers of bacterial evolution and worldwide health risks. Recognizing plasmids as connectors across microbiomes highlights both the urgency and opportunity to address plasmid-mediated resistance with integrated strategies. Current plasmid databases, such as NCBI RefSeq, PLSDB, IMG/PR, and PlasmidScope, have already greatly advanced our understanding of these connections, and they are likely to profoundly alter how we see plasmid biology and One Health relationships.

Plasmids are extrachromosomal DNA molecules that are often present in prokaryotic cells but replicate independently of their host chromosome. They are widely distributed across diverse bacterial and archaeal species and habitats (Figure 1). They are key agents in bacterial evolution through horizontal gene transfer (HGT), contributing to adaptation and genetic diversity. They often carry genes that provide their hosts with selective advantages, such as antibiotic resistance, virulence, and tolerance to heavy metals, enabling the host to survive in adverse conditions [1,2]. A recent study with 2045 genomes of *Escherichia coli* has shown that plasmids act as evolutionary enablers in *E. coli*, carrying genes that influence whether a clone thrives or fades away. Some successful *E. coli* clones owe their dominance to antibiotic resistance, but others owe it to plasmid-encoded tools like bacteriocins, which help them compete in microbial ecosystems such as the human gut [3]. For a review on the origin and history of plasmids, see [4]. This review focuses on the diversity, classification, mobility, and functional roles of plasmids, particularly in the context of antibiotic resistance and virulence gene dissemination. It discusses how plasmids act as key mobile genetic elements shaping bacterial evolution, with strong implications for public health and the One Health approach.

## 1. The Ubiquity of Plasmids

**Figure 2:**
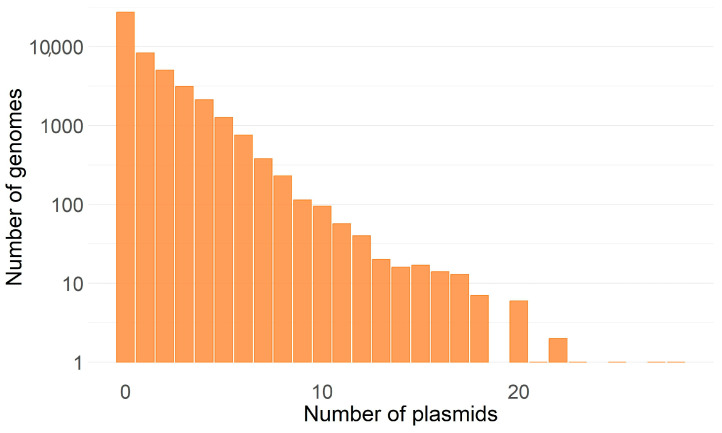
Distribution of the number of prokaryotic genomes (bacteria and archaea) with a given number of plasmids. The number of genomes (vertical axis) is on a Log10 scale, and the horizontal axis is a linear scale. Data analyzed according to [5].

Last February, the NCBI RefSeq database contained 49,114 non-redundant complete prokaryotic genomes, comprising 7715 host species of bacteria and archaea. Of these, 20,580 (41.9%) had plasmids, and the other 64.9% of the genomes had no identified plasmids [5]. On average, each genome contains 1.1 plasmids or 2.6 if we only consider genomes with plasmids (Figure 2). While many genomes have few plasmids, others have many. This enables the exchange of genetic information between plasmids, which can then be spread to other bacteria (Figure 2).

Table 1 summarizes the number of genomes in the RefSeq database containing the highest number of plasmids, considering species with more than 1 and up to 10, more than 10 and up to 100, and more than 100 complete genomes (Table 1). In each of these analyses, the species with more plasmids per genome are *Arsenophonus nasoniae* (15.7 plasmids), *Borreliella burgdorferi* (14.4 plasmids), and *Enterococcus faecium* (3.3 plasmids).

In species without plasmids, the question inevitably arises as to whether the low number of samples means that plasmids are not being detected. However, for some species, several strains were sequenced, yet no plasmids were detected: for example, in the 672 genomes of *Bordetella pertussis*, 443 of *Mycobacterium tuberculosis*, 160 of *Corynebacterium pseudotuberculosis*, and 122 of *Neisseria meningitidis* [5]. This considerable number suggests that these species naturally lack plasmids.

In contrast, a *Chondrinema litorale* strain WSW3-B12 was isolated from a red algae collected in the Republic of Korea [6], containing 28 plasmids. Another striking case is from the species *Borrelia coriacea* strain Co53, which was isolated from the tick *Ornithodoros coriaceus* [7] and harbors 23 complete plasmids. These strains are the only ones sequenced of the respective species, so they were not included in Table 1.

It is remarkable that a single cell lineage can maintain so many different plasmids. To maintain themselves in bacterial cells and their vertical lineages, plasmids must regulate their copy number. If there are two or more different plasmids in the same bacterium, the copy-number control of one must not ‘count’ copies of the other plasmids. In other words, the two or more plasmids present in a bacterial lineage must be sufficiently different to be compatible. This was the basis of one of the main ways to classify plasmids, as we discuss in the next section.

## 2. Plasmid Classification

Due to their genomic flexibility, plasmids are difficult to classify. The way they are classified has evolved since their discovery in the 1940s. The first plasmid identified was the F plasmid, which was discovered in *E. coli* by Lederberg and Tatum [8]. This discovery was fundamental to the understanding of bacterial recombination mechanisms, as it revealed that bacteria transfer genetic material through conjugation. Subsequently, in the late 1950s, R plasmids were discovered. These plasmids carry genes responsible for antibiotic resistance. Another important group identified was the Col (colicinogenic) plasmids. These plasmids encode bacteriocins, toxins that kill or inhibit the growth of closely related bacteria. The presence of Col plasmids, therefore, gives bacteria a competitive advantage, enabling them to dominate specific ecological niches. Further research has shown that R and Col plasmids are similar to common plasmids, although carrying genes coding for resistance or bacteriocins thanks to microevolution and fusion events [9,10,11]. Subsequently, plasmids began to be classified into incompatibility groups (Inc) based on the ability to coexist in the same bacterial cell. Similar plasmids, i.e., plasmids of the same Inc group are unable to be maintained in the same host cell [12]. These groups are based on the stability of plasmid-borne bacteria when other plasmids are present. That is, this classification separates plasmids into groups that cannot stably coexist in the same bacterium. This is due to their similar replication systems, which can lead to interference, resulting in the loss of one plasmid during cell division—that is, the regulatory system counts the copies of the other plasmid as of their own [13,14,15]. As a consequence, at least one of the plasmid types does not replicate as much as it should. During cell division, that plasmid may fail to be inherited by the next bacterial generation due to incorrect segregation. These plasmid types (the one that is lost and one of the plasmid types that is not lost) are said to be incompatible. Attempts to classify plasmids into different incompatibility groups involved introducing a new plasmid into a host cell carrying another plasmid and assessing whether both could be stably maintained [16,17]. However, this method was time-consuming and labor-intensive, limiting its application to a small number of plasmids.

Advances in molecular biology techniques, such as PCR, have enabled the rapid detection of specific replicons (autonomous replicating DNA molecules) for each Inc group [18], facilitating the fast and large-scale identification of these replicons [19]. The replication mode of a plasmid determines its compatibility with other plasmids within the same cell. But classification by incompatibility groups includes plasmids with large genetic differences; that is, two plasmids may belong to the same Inc group (because they share the same replication system) but differ significantly otherwise [20].

Since genetic similarity may not exist within the same incompatibility group, and due to advances in sequencing power resulting in an increase in available genetic data, other methods have been developed. One of the methods is MOB typing, which is based on mobility genes [19,21,22].

MOB typing is a particularly relevant approach because it is based on relaxases, which are enzymes fundamental to plasmid transfer by conjugation. These proteins initiate the transfer of plasmid DNA by cleaving the origin of transfer (*oriT*)—the site of initiation of DNA transfer in plasmid-mediated bacterial conjugation—and their presence is a key indicator of a plasmid’s mobilization potential. Relaxases are relatively conserved within groups of evolutionarily related plasmids, which allows them to be used as robust phylogenetic markers [21]. Each MOB family (e.g., MOBF, MOBH, MOBP and MOBQ) represents a distinct evolutionary lineage of relaxases. Classification based on these families enables the inference of ancestral relationships. This approach is helpful in practice, for example, in epidemiological surveillance of plasmids that carry antibiotic resistance genes. However, MOB typing has limitations, particularly regarding non-conjugative plasmids (pNT), where mobilization genes may be absent. It can also be challenging to assign recombinant plasmids containing multiple relaxases to a single lineage.

Another method to classify plasmids is based on shared k-mer content [23]. With this approach, plasmids are grouped according to the similarity of their genetic sequences. K-mers are fixed-length (k) DNA sequences that can be used to measure the distance or similarity between genomes or genomic elements, such as plasmids. Comparing the frequency and presence of these k-mers across different plasmids enables the inference of evolutionary and taxonomic relationships, thereby overcoming the limitations of traditional methods that focus solely on specific genes or restricted markers [23]. K-mer analysis is a fast, computationally efficient approach that is useful for initial screening or working with incomplete data; however, it may lack adequate resolution.

Advances in next-generation sequencing and bioinformatics methodologies have made more robust techniques available, allowing the development of a classification system based on the overall genetic similarity, such as Plasmid Taxonomic Units (PTUs), similar to the concept of operational taxonomic units previously developed for bacteria [24,25]. The classification of plasmids into PTUs requires the use of the Average Nucleotide Identity (ANI) and the Alignment Fraction (AF). These units offer a systematic, species-independent approach, enabling plasmids to be classified based on genomic distances rather than just the presence of specific genes. Plasmids are organized into PTUs based on two criteria related to the similarity of their genetic sequences. First, the two plasmids must align at least 50% of the length of the shorter plasmid. Second, the ANI value between them must be equal to or greater than 70% [24]. If these two criteria are met, the two plasmids belong to the same PTU. This methodology is incorporated in the COPLA software, which automatically assigns plasmids to known PTUs or identifies possible new PTUs [25]. In addition to sequence similarity, PTUs often exhibit shared characteristics, such as a conserved set of core genes, despite variations in accessory genetic content [26].

Meanwhile, reference-free approaches have emerged, such as MGE-cluster, which groups plasmids based on genetic similarity, independent of previously known replicons or annotated databases [27]. MGE-cluster works by decomposing plasmid sequences into unitigs. A unitig is a continuous sequence obtained from an assembly graph, consisting of sections without branches where the sequence is unambiguous. Based on these sequences, a presence-absence matrix is constructed, and dimensionality reduction and clustering are applied to identify groups of genetically related plasmids. In this way, it captures both known and novel plasmid backbones, facilitates the detection of resistance-carrying plasmids, and provides a scalable framework for plasmid classification. This technique is helpful in identifying emerging strains or poorly studied plasmids, making it ideal for analyzing metagenomic data or environmental isolates [27].

Despite these advances, plasmid classification continues to present significant challenges due to the high frequency of plasmid recombination, the absence of highly conserved genes and the constant acquisition of mobile genetic elements. To overcome these limitations, integrative approaches combining MOB typing, replicon typing, PTU and MGE-cluster can be used. These strategies enable more accurate plasmid classification and facilitate the inference of evolutionary history and patterns of horizontal dissemination.

## 3. The Influence of Plasmid Mobility on Gene Dissemination

Plasmid mobility is a fundamental aspect of genomic dynamics and evolution, not only of plasmids themselves, but also of the chromosomes. It contributes significantly to the spread of adaptive traits, such as genes associated with antibiotic resistance and virulence [22,28]. In terms of mobility, plasmids can be divided into three main categories: conjugative (pCONJ), mobilizable (pMOB), or non-transmissible (pNT).

pCONJs encode all the necessary genes for self-transfer between two bacteria. These include relaxases, which initiate plasmid transfer at the origin of transfer (*oriT*); type IV coupling proteins (T4CP); and type IV secretion systems (T4SS). These systems enable the formation of a conjugative pilus, which establishes physical contact between the donor and recipient cells [29,30,31]. pMOBs, on the other hand, lack part of the conjugation machinery. They contain relaxases and *oriT* and utilize the conjugation systems encoded by other pCONJs present in the same cell for transfer [22,32]. Plasmids harboring an *oriT* but lacking relaxases are also mobilizable. These plasmids, called pOriT, typically exploit more than one plasmid to facilitate their transfer, using the conjugation systems of one plasmid and the relaxase of another [29]. pNTs cannot be transferred by conjugation because they lack an *oriT* and the transfer genes necessary for conjugation. Some non-transmissible plasmids already identified may actually be pOriT, as origins of transfer are currently not well understood [29,33].

Recent studies suggested that the type of mobility of plasmids may change due to their high genetic plasticity. pCONJs may evolve to pMOBs, while pCONJs/pMOBs may lose their ability to be transferred, becoming pNTs [34,35]. Coluzzi et al., analyzed the relaxases of over 11 thousand plasmids to evaluate changes in plasmid mobility [34]. DNA regions encoding relaxases were chosen because they demonstrate significant genetic conservation. The relaxases found in pMOBs are distinct from those present in pCONJs. Analysis of plasmids harboring relaxases revealed cases where relaxases related to pMOBs were found in pCONJs, and vice versa. Such cases suggest that shifts in mobility type have occurred relatively recently. Interestingly, pCONJ containing pMOB-type relaxases are less common than the opposite, indicating a higher probability of transitions from pCONJ to pMOB than vice versa. Hanke et al. reinforced the evidence of mobility changes by studying pseudogenes [35]. Pseudogenes are non-functional gene homologues that are unable to encode active proteins. The authors found that pseudogenes occur more frequently in plasmids than in chromosomes, with the majority located in regions previously associated with plasmid mobility. They also observed that larger plasmids with a higher number of pseudogenes tend to be mobilizable or non-transmissible, with these pseudogenes often concentrated in mobility-associated regions. This suggests that large plasmids, comparable in size to pCONJ, have altered their mobility capacity. When a pseudogene compromises mobility, the probability increases that the remaining genes involved in the process will also lose their function [35].

Due to their mobility, plasmids can be found in bacteria belonging to very different taxa. Therefore, in terms of host range, plasmids can be either broad- or narrow-ranging. Broad-range plasmids can replicate in a wide variety of bacterial taxa, while narrow-range plasmids can only replicate in a small number of species, usually within the same genus. Recently, Redondo-Salvo et al. (2021) proposed a six-level scale to classify the host range of plasmids, ranging from grade 1 (restricted to a single species) to grade 6 (capable of replicating in different bacterial phyla) [25].

The mobilization of plasmids plays a critical role in spreading ARGs and virulence genes (VGs) among pathogenic bacteria via HGT. This mechanism significantly contributes to the emergence of multidrug-resistant strains with greater pathogenic potential, posing an increasing threat to public health [36,37]. This occurs because pCONJs and pMOBs facilitate not only the spread of plasmids but also the dissemination of virulence factors, thereby increasing the virulence of recipient bacteria [24,38]. In addition, plasmids often interact with other mobile genetic elements, including transposons, integrons, and insertion sequences, which promotes the reorganization and combination of multiple genetic determinants of resistance and virulence [39] and facilitates the evolution of plasmids.

## 4. Plasmids as Drivers of Antibiotic Resistance

Antibiotic resistance, one of the greatest threats to public health, is partly an ancestral and natural phenomenon [40]. D’Costa et al. analyzed DNA from permafrost sediments (approximately 30,000 years old) to search for genes that confer resistance to antibiotics used nowadays. They detected multiple ARGs commonly associated with current antibiotic resistance in these samples, such as β-lactams and vancomycin. Functional and structural characterization of the *vanA* gene revealed many similarities with modern variants. These results suggest that antibiotic resistance is not exclusively a modern phenomenon and is not solely caused by clinical use of antibiotics [40].

However, several studies indicate that the use of antibiotics in clinical and agricultural contexts promotes the emergence and selection of antibiotic resistance-encoding genes [41,42]. Continuous exposure to antibiotics exerts selective pressure that favors microorganisms carrying resistance genes, which can spread among different bacterial species through horizontal gene transfer mechanisms such as conjugation (see the section related to plasmid mobility). This contributes to the emergence of multidrug-resistant strains, which pose a growing threat to public health [43,44,45]. For example, pathogens such as *E. coli* and *K. pneumoniae* often carry multiple ARGs, which limit available therapeutic options [46]. Hussaini et al. (2024) used two *E. coli* strains and four *K. pneumoniae* strains, which are resistant to β-lactam antibiotics once they carry the *blaNDM*, *blaOXA*, or both genes, to test their susceptibility to other antibiotics [47]. They found that all the strains were resistant to trimethoprim-sulfamethoxazole, and exhibited high resistance to doxycycline (83.33%), chloramphenicol (66.67%), and nalidixic acid (66.67%).

Some environments, such as wastewater from clinical and agricultural sources, exhibit significantly greater diversity and abundance of ARGs than unexposed environments [48,49]. Exposure to a specific antibiotic tends to reduce the diversity of resistance genes to other antibiotics, while increasing the abundance of genes conferring resistance to the administered antibiotic [50]. In samples receiving waste from multiple sources—such as hospital wastewater, which collects residues from many patients—there is a mixture of bacteria carrying different resistance genes, whose abundance may reflect the different antibiotics used. Therefore, while exposure to a single antibiotic increases the abundance of specific resistance genes, contamination from diverse sources exposed to different antibiotics can lead to greater overall diversity of resistance genes. Zhu et al. compared samples of manure from pigs exposed to antibiotic treatment and fertilized soil, and from pigs not exposed to antibiotics and unfertilized soil. The 63 most frequent resistance genes were 192 to 28,000 times more abundant in environments exposed to antibiotics than in environments where these drugs were absent. Additionally, the diversity of resistance genes was much higher in samples from sources exposed to antibiotics [49].

Moreover, a direct link exists between environmental reservoirs and clinical environments [51,52,53,54,55,56]. Lee and colleagues collected samples from a South Korean river to investigate the presence of ARGs. Samples were collected at various locations along the river and at different times of the year [57]. Through metagenomic analysis, they searched for ARGs and human pathogens to establish an association between environmental and clinical reservoirs. They identified genes encoding resistance to aminoglycosides, sulfonamides, β-lactams, tetracyclines, amphenicols, macrolides, lincosamides, and streptogramin B, which had a high capacity for HGT. A strong correlation was also identified between the abundance of ARGs and the presence of human pathogenic bacteria in the samples. These evidences highlight the urgent need for policies on the rational use of antibiotics and for the monitoring of antimicrobial resistance in the environment.

There are two important classifications of pathogenic bacteria, according to their level of resistance to antibiotics. According to the World Health Organization (WHO), pathogens are divided into different priority levels to identify the resistant bacteria that pose the greatest threat to public health and require urgent responses in terms of research, development, and health policies. To this end, each pathogen is evaluated according to a set of criteria that reflect both the severity of the disease it causes and the difficulty of controlling and treating it [46].

The other categorization is related to the most important pathogenic bacteria, notably the group of seven bacterial species known as ESKAPEE. Initially, this group was defined as ESKAPE, comprising the six pathogens *Enterococcus faecium*, *S. aureus*, *K. pneumoniae*, *Acinetobacter baumannii*, *Pseudomonas aeruginosa*, and *Enterobacter* species [58]. Later, *E. coli* was added to the group, which became known as ESKAPEE [59]. These bacterial pathogens cause infections that can “escape” or resist the most common therapies due to their ability to develop resistance to antibiotics.

The presence of ARGs in plasmids enhances their dissemination among different bacterial species when antibiotics are present. This accelerates the spread of resistance in microbial populations and significantly expands the epidemiological reach of resistance [22,38,60]. Many plasmids carry resistance islands, which are clusters of ARGs that are often integrated into mobile genetic elements, such as transposons and integrons [38]. These genetic structures enable the simultaneous transmission of resistance to several classes of antibiotics, thereby exacerbating the problem of multidrug resistance. A study of more than 16,000 closed bacterial genomes [61] revealed that, on average, each genome (including the bacterial chromosome and plasmids) encodes resistance to approximately nine different antibiotic classes, indicating a concerning level of multidrug resistance. There are significantly higher co-occurrences than expected for several combinations of antibiotic classes, including those involving β-lactams, glycopeptides, quinolones, macrolides, and colistin with other antibiotic classes. In WHO priority pathogens, ARGs co-occurrences are predominantly located on plasmids rather than chromosomes, suggesting a recent and epidemic acquisition [61].

**Figure 3:**
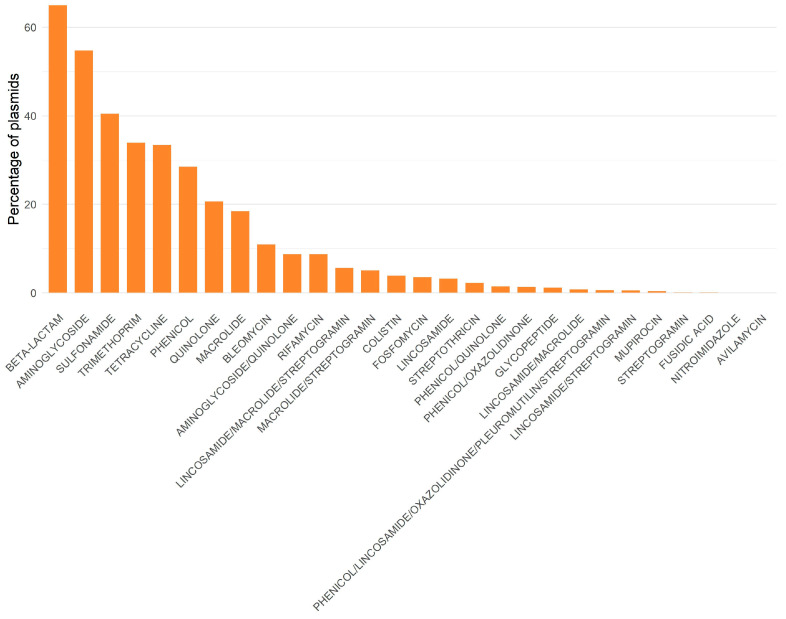
Percentage of plasmids in which each class of antibiotic resistance is present. Data analyzed according to [5].

Another study, involving almost 53,000 complete, non-redundant plasmids obtained from RefSeq (unpublished results), identified resistance to 28 classes of antibiotics. Resistance genes to beta-lactams, aminoglycosides, and sulfonamides were found in over 40% of the plasmids (Figure 3). More than half of the plasmids analyzed contained β-lactam and aminoglycoside. This is concerning, given that these antibiotics are of great importance in both human and veterinary medicine. Many co-occurrences among different resistance classes occurred more frequently than expected (for example, aminoglycosides with macrolides, β-lactams with aminoglycosides, and β-lactams with macrolides), indicating that there may be co-selection of different classes inside each plasmid (unpublished results). Moreover, there are interesting differences between plasmids found in ESKAPEE and non-ESKAPEE bacteria: plasmids not belonging to the ESKAPEE group exhibit more co-occurrences than expected (unpublished results).

Regarding co-occurrences by mobility type, we observed that pCONJs exhibited more co-occurrences than expected compared to pMOBs and pNTs. One might consider this result expected because conjugative plasmids can move between different bacterial cells, sometimes between different bacterial taxa, as we discussed above. Moreover, pCONJs are expected to be exposed to antibiotics more often, leading to the selection and accumulation of different resistances. Thirdly, these plasmids are larger, which may explain why they have resistance genes for more antibiotic classes. One might establish similar comparisons between pMOBs and pNTs. However, pMOBs have fewer co-occurrences than pNTs (unpublished results). This last result is counterintuitive, since pMOBs circulate between hosts, making them more likely to acquire different genes. Additionally, in the face of the works discussed above showing pCONJs tend to evolve towards pMOBs and these into pNTs [34,35]. The co-selection of different antibiotics is alarming from a public health perspective, particularly when it involves last-resort antibiotics used as a final therapeutic option. Co-occurrences between resistance genes to commonly used antibiotics and those conferring resistance to last-resort ones can lead to the unintended selection and spread of multidrug-resistant determinants, even in the absence of direct selective pressure. Therefore, monitoring these co-occurrences is essential when surveying antimicrobial resistance. In particular, co-occurrences in plasmids require special attention, as they can rapidly disseminate resistance genes across bacterial populations. Integrating co-occurrence analysis into clinical microbiology diagnostics, veterinary antimicrobial stewardship, and epidemiological surveillance programs could help identify emerging resistance threats earlier and guide targeted interventions to limit their spread.

The result that pCONJs harbors more co-occurrences of ARGs is consistent with the results of Coluzzi et al. (2025), who analyzed over 37,000 plasmids [62]. Of these, 9384 (approximately 25%) encoded at least one antibiotic resistance gene, covering 392 unique genes distributed across 31 classes of antibiotics. The plasmids exhibited 895 distinct combinations, with a median of three classes per plasmid. The authors then grouped the plasmids into 355 PTUs. Analyzing the PTUs containing plasmids encoding resistance, Coluzzi et al. (2025) demonstrated that PTUs with ARGs tend to include pCONJs [62]. These PTUs have plasmids with a broader host range, i.e., they are present in more distinct host taxa, and exhibit higher rates of homologous recombination in core genes and larger pangenomes than PTUs without ARGs. These patterns remained when plasmids containing ARGs were removed from the PTUs, indicating that plasticity and mobility are inherent traits of these plasmid units. They function as critical vectors in the horizontal transfer of resistance genes between bacteria from different biomes and taxonomic groups.

## 5. Plasmids and Virulence Genes

The coexistence of both ARGs and VGs in bacterial pathogens may pose a significant threat to public health. Unfortunately, in addition to the co-selection of genes from two antibiotic resistance families, it is possible that ARGs are also co-selected alongside VGs. There are, however, three levels to consider: the metagenome, the bacterial cell, and replicons (chromosomes or plasmids).

In human metagenomes, the diversity of ARGs and VGs is positively correlated [63], meaning that metagenomes with higher antibiotic resistance also tend to have a greater number of VGs. This has been observed in metagenomes from Mali, Venezuela, and the USA. In that work, the authors reasoned that taking antibiotics while infected with pathogenic bacteria would select for bacterial cells that are simultaneously antibiotic-resistant (hence with ARGs) and pathogenic (hence with VGs). However, that explanation might not work because antibiotics are expected to target multiple strains and species of bacteria, not just pathogens [64].

Computer simulations of a network of people interacting with each other and infecting each other have given rise to another explanation for the positive correlation between the number of ARGs and VGs in metagenomes [50]. These simulations have demonstrated that metagenomes can accumulate virulence and antibiotic resistance genes over extended periods. When some infected individuals are treated with antibiotics, the susceptible bacteria are eliminated while the resistant cells survive. These surviving cells are resistant to the administered antibiotics and may also be resistant to other antibiotics. However, the eliminated cells have a high diversity of ARGs and VGs, which results in a general reduction in the diversity of these genes in the metagenome. Individuals who have recently received antibiotics exhibit the lowest diversity of both resistance and virulence genes, whereas those treated in the more distant past show the highest diversity for both gene types—resulting in a positive correlation across human metagenomes [50]. Interestingly, this implies that individuals who took antibiotics a long time ago are the ones with more diversity of ARGs and VGs [50].

The simulations suggest something else: the positive correlations between ARGs and VGs observed at the metagenome level may not exist at the level of individual cells. Comprehensive genomic analysis of bacterial plasmids and chromosomes corroborates this expectation, showing that there is no correlation at the bacterial genome level [65]. Individual bacterial cells with more ARGs are not necessarily those with more VGs. Results concerning only the chromosome or only plasmids yielded the same qualitative result: no correlation was found between the number of ARGs and VGs [65].

Although there is no correlation at the genomic level, the same work demonstrated that certain resistance families have a higher-than-expected frequency of co-occurrence in the same genome, particularly when located in plasmids. The selective pressure exerted by antibiotic use favors not only the maintenance of resistance but also indirectly stabilizes some virulence determinants. This increases the severity of infections and drastically reduces therapeutic options [65].

## 6. Influence of Plasmid Data Origin on Research Outcomes

There are several repositories of plasmid data, allowing them to be characterized and studied. Examples of plasmid repositories include the Reference Sequence Database (RefSeq) [66], PLSDB [67,68], IMG/PR [69] and PlasmidScope [70].

The RefSeq database is a curated, non-redundant collection of genomic, transcriptomic, and protein sequences maintained by the National Center for Biotechnology Information (NCBI). It includes plasmid sequences derived from various bacterial species. The plasmids are associated with metadata containing information about the year and the environment from which the sample was collected, the host taxonomy, and the geographic location. However, as the sample depositor manually inserts these values, this data is not always in a standard format when available, which complicates processing and analysis. This database is constantly updated. For reference, the RefSeq database contained 38,057 complete plasmids in 2023 [62] and 54,264 complete plasmids in 2025 [5]. This represents a 42% increase in the number of complete plasmids deposited in the database over a two-year period.

PLSDB is also a curated plasmid database that includes complete plasmid sequences from a broad range of bacterial species. It integrates data from RefSeq and other databases of the International Nucleotide Sequence Database Collaboration (INSDC) and currently contains over 72,000 complete plasmid sequences. The metadata in PLSDB includes information on host taxonomy, geographic and ecological origins, plasmid similarities, and functional elements. As this database contains data from various sources, the metadata may differ between plasmids.

The IMG/PR database is a plasmid repository integrated into the Integrated Microbial Genomes & Microbiomes (IMG/M) platform. It offers a comprehensive collection of plasmid sequences identified from thousands of isolated genomes, single amplified genomes (SAGs), and metagenome-assembled genomes (MAGs), as well as metagenomes and metatranscriptomes. This database contains both putatively complete and incomplete sequences. Associated metadata includes sample origin, host taxonomy, the presence of resistance and mobility genes. However, the taxonomic information is particularly incomplete for metagenomic data due to the difficulty of associating plasmids with their hosts, and even when the host is detected, the difficulty in identifying the host’s taxonomy remains.

PlasmidScope is a database comprising more than 800,000 unique plasmid sequences. It contains sequences from ten different repositories, including RefSeq, PLSDB, IMG/PR, and GenBank. This database aims to consolidate all existing databases, providing a uniform gene annotation pipeline for all plasmids. This indicates the functions detected for all plasmids, including those from the Kyoto Encyclopedia of Genes and Genomes (KEGG) and Clusters of Orthologous Groups (COG).

Plasmids from different databases were isolated and sequenced using various methods, as previously described. Recently, a comprehensive analysis of plasmids from three distinct datasets was conducted, plasmids from the RefSeq database, isolate plasmids from the IMG/PR database, and plasmids sequenced from metagenomes from the IMG/PR database, to understand the differences between the datasets [71]. This distinction is essential because analyzing, identifying and assembling plasmids from isolates or metagenomes involve different processes and are subject to various errors and biases. Detecting and assembling plasmids from bacterial isolates and metagenomic data remains a major challenge due to their high variability in size and copy number, as well as the presence of highly repetitive regions similar to chromosomal DNA [72,73]. This complexity increases the likelihood of fragmented or erroneous assemblies, particularly for large, low-abundance plasmids whose coverage is equivalent to that of the bacterial genome [72]. The limitations of sequencing data also contribute to these difficulties. Despite their high accuracy, short reads often yield fragmented assemblies, particularly in repetitive or complex regions of plasmids. In addition to the common problems associated with assembling and identifying plasmids from isolates and metagenomics, metagenomes present additional challenges. These samples contain DNA from different host species. Therefore, similar plasmids from different hosts may be assembled as a single plasmid. Furthermore, plasmids with a low copy number and abundance in hosts may go unidentified. Even when they are identified, it is difficult to link them to their hosts. Long-read technologies enable the generation of more complete plasmid sequences, but they also introduce higher error rates. They may fail to detect small or low-abundance plasmids, thereby limiting their sensitivity in microbial communities.

Recent results revealed that the proportion of plasmid mobility types is strongly dependent on the database [71]. Data from RefSeq revealed a higher frequency of pCONJs, which tend to be larger and more likely to encode ARGs or VGs. In contrast, plasmids assembled from metagenomes are enriched in pMOBs, which are smaller and, on average, have fewer ARGs and VGs. However, this may be due to difficulties in detecting and assembling plasmids in metagenomes rather than to differences between clinical and environmental samples. The distinction between metagenomes and isolates is critical as it shows that conclusions about the functional and epidemiological distribution of plasmids can be affected by the type of dataset being studied.

Comparing different data sources reveals that the prevalence of pCONJs, pMOBs, pNTs, and pOriTs is strongly influenced by the origin of the sample and the sequencing method used. Studies based exclusively on clinical isolates are possibly biased and likely overestimate the importance of pCONJs. In contrast, more comprehensive metagenomic analyses detect a greater diversity and abundance of pMOBs and pNTs. These variations have significant implications for genomic surveillance and modeling the spread of antibiotic resistance and reinforce the need to carefully consider the origin of the data when interpreting results on plasmid mobility. Different types of mobility are associated with distinct functional profiles, and this association is, in great part, influenced by the dataset analyzed. Additionally, when comparing the sizes of pCONJ, pMOB, and pNT, plasmids in each category are consistently larger in the RefSeq database, followed by IMG/PR(I) and then IMG/PR(M). In other words, the average size of plasmids in each category is even influenced by the database being analyzed.

To ensure robust and generalizable interpretations, it is crucial to integrate data from multiple sources, namely clinical, environmental, and metagenomic sources, and adopt standardized criteria for plasmid classification. This should include the systematic identification of origins of transfer, which may reveal hidden mobility in plasmids that were previously considered non-transmissible.

## 7. Plasmids and the One Health Concept

The fact that plasmids act as mobile vectors of antibiotic resistance genes that circulate between different bacterial species and ecosystems reinforces the relevance of the One Health concept, which recognizes the intrinsic link between human health, animal health, and the environment [74]. Plasmid-mediated genetic mobility reveals that resistance is not an isolated problem in hospital environments but rather a global phenomenon driven by the inappropriate or excessive use of antimicrobials in various contexts, including healthcare, agriculture, aquaculture, and livestock farming.

The risk to public health is even greater when these bacteria carrying ARGs are also pathogenic. In such cases, they combine the ability to colonize and infect hosts with resistance to one or more antibiotics, potentially causing severe infections that may be difficult to treat. However, the administration of antibiotics also has other, less obvious consequences. When an antibiotic is administered to treat an infection, all sensitive bacteria, whether pathogenic or commensal, are potentially equally affected [64]. This creates an imbalance that favors the survival and dissemination of resistant strains, with non-pathogenic bacteria serving as silent reservoirs of ARGs. These ARGs can later be mobilized to pathogenic bacteria due to the selective pressures exerted by antibiotic use.

**Figure 4:**
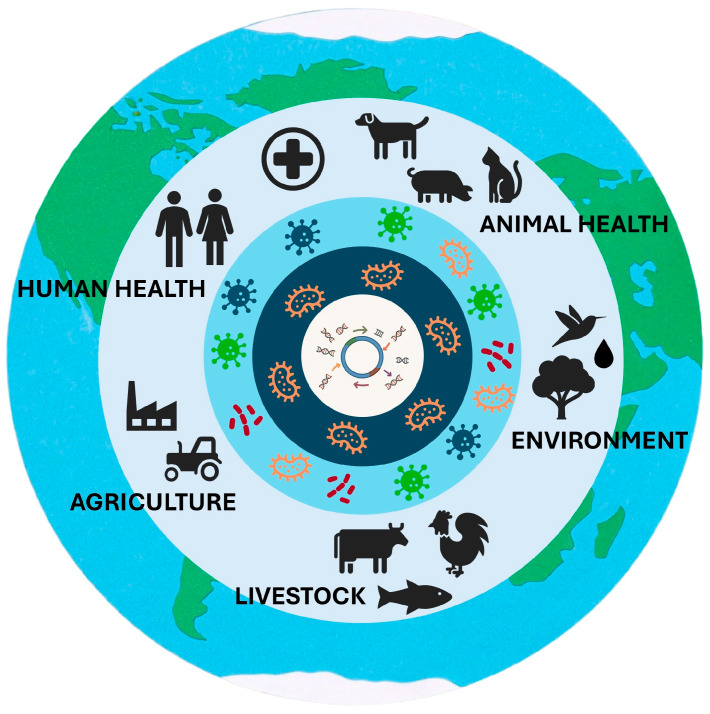
The genetic dynamics of plasmids are intertwined with those of bacterial species and communities, and with the connections linking human, animal, and environmental contexts. This image illustrates the various levels of interaction, ranging from the microscopic to the planetary.

Plasmids, owing to their high genetic plasticity and dynamic nature, are highly mobile elements capable of crossing multiple biological and ecological barriers. They readily acquire or lose genes through recombination with other mobile genetic elements or with the bacterial chromosome. Beyond intra-population transfer, plasmids can move between pathogens or between pathogens and commensal bacteria; the latter often serve as reservoirs of genes conferring antibiotic resistance, tolerance to toxic compounds, or virulence traits. Broad-host-range plasmids are particularly adept at crossing species and even higher taxonomic boundaries. Moreover, plasmids can disseminate between microbial communities through bacterial exchange across different microbiomes, enabling their circulation among humans, animals, and the wider environment, spanning air, soil, and water, which further accelerates plasmid evolution and enhance their genetic plasticity (Figure 4). For example, in aquatic environments, plasmids from *E. coli* isolates harbored the *mcr-1* gene, which was associated with strains of *E. coli* from human and animal origins [75]. More recently, other variants of the *mcr* gene have been identified in bacteria isolated from humans, animals, food, and the environment in various geographical locations [76]. These cases demonstrate the ability of plasmids to spread between different environments.

Within the One Health framework, this is particularly concerning, as non-pathogenic bacteria are widespread across humans, animals, and environmental niches, facilitating gene flow between ecosystems. This interconnectedness highlights that controlling antimicrobial resistance requires not only monitoring pathogenic species but also addressing the role of plasmids in the genetic exchange potential of the broader microbial community, which links human, animal, and environmental health, as advocated by the One Health perspective.

## 8. Conclusions

With their dynamic genomes, plasmids are key drivers of bacterial evolution, facilitating the spread of antibiotic resistance and virulence factors across species and ecosystems. Their plasticity and mobility allow for the dissemination of adaptive traits, impacting entire microbial communities. Variability in data sources further shapes our understanding of plasmid functions, underscoring the need for reliable environmental data to characterize plasmids accurately. From a One Health perspective, plasmids act as global connectors of resistance and virulence, emphasizing both their epidemiological significance and the urgent need for integrated surveillance strategies.

## Figures and Tables

**Figure 1:**
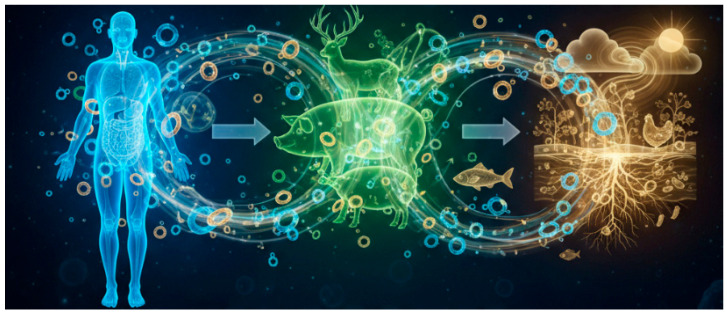
Plasmids circulate across human, animal, and environmental contexts, facilitating the horizontal transfer of genetic material. These mobile elements frequently carry antibiotic resistance and virulence genes, which can coexist within the same plasmid. This genetic linkage enhances the risk of co-selection. Image generated with artificial intelligence (https://deevid.ai/, accessed on 10 October 2025).

**Table 1:** Species with the highest number of genomes in the RefSeq database and plasmids per genome, considering those with more than 1, 10, or 100 genomes in the database. Data analyzed according to [5].

	Species	Number of Genomes	Number of Plasmids per Genome
1 < Nr. genomes < 10	*Arsenophonus nasoniae*	6	15.7
	*Borreliella carolinensis*	2	15.0
	*Borreliella japonica*	2	15.0
	*Borreliella kurtenbachii*	2	15.0
	*Borreliella bissettiae*	2	13.5
10 < Nr. genomes < 100	*Borreliella burgdorferi*	29	14.4
	*Pseudosulfitobacter pseudonitzschiae*	43	6.1
	*Acinetobacter lwoffii*	13	5.8
	*Bacillus thuringiensis*	97	5.5
	*Borrelia miyamotoi*	43	5.0
Nr. genomes > 100	*Enterococcus faecium*	338	3.3
	*Klebsiella pneumoniae*	2627	3.0
	*Klebsiella quasipneumoniae*	151	2.9
	*Enterobacter hormaechei*	398	2.8
	*Shigella flexneri*	112	2.7

## Data Availability

Not applicable.
